# The prevalence of, and factors associated with, paying for sex among men resident in Britain: findings from the third National Survey of Sexual Attitudes and Lifestyles (Natsal-3)

**DOI:** 10.1136/sextrans-2014-051683

**Published:** 2014-11-17

**Authors:** Kyle G Jones, Anne M Johnson, Kaye Wellings, Pam Sonnenberg, Nigel Field, Clare Tanton, Bob Erens, Soazig Clifton, Jessica Datta, Kirstin R Mitchell, Phillip Prah, Catherine H Mercer

**Affiliations:** 1 Research Department of Infection & Population Health, University College London, Mortimer Market Centre, London, UK; 2 Department of Social and Environmental Research, London School of Hygiene & Tropical Medicine, London, UK; 3 Department of Health Services Research and Policy, London School of Hygiene & Tropical Medicine, London, UK; 4 NatCen Social Research, London, UK

**Keywords:** Sexual Behaviour, Sexual Health, Social Science

## Abstract

**Objectives:**

Men who pay for sex (MPS) are considered a bridging population for sexually transmitted infections (STI). However, the extent, characteristics and role of MPS in transmission is poorly understood. We investigate these questions using data from Britain's third National Survey of Sexual Attitudes and Lifestyles (Natsal-3).

**Methods:**

We performed complex survey analyses of data from 6293 men aged 16–74 years resident in Britain who completed Natsal-3, a probability sample survey undertaken during 2010–2012, using computer-assisted personal interviewing and computer-assisted self-interview.

**Results:**

11.0% (95% CI10.1% to 11.9%) of all men reported ever paying for sex. Among MPS, 18.4% (95% CI 18.2% to 18.7%) of their lifetime sexual partners were paid. 3.6% (95% CI 3.1% to 4.2%) of men had paid for sex in the past 5 years. Partners of MPS constitute 14.7% of all reported partners and MPS report 15.6% of all reported STI diagnoses in the past 5 years. Paying for sex in the past 5 years was strongly associated with reporting larger numbers of sexual partners (adjusted OR, AOR for 5+ partners, past 5 years, 31.50, 95% CI 18.69 to 53.09). After adjusting for partner numbers, paying for sex remained strongly associated with reporting new foreign partners outside the UK (AOR 7.96; 95% CI 4.97 to 12.73) and STI diagnosis/es (AOR 2.34; 95% CI 1.44 to 3.81), all in the past 5 years. Among men ever paying for sex, 62.6% (95% CI 58.3% to 66.8%) reported paying for sex outside the UK, most often in Europe and Asia.

**Conclusions:**

MPS in Britain remain at greater risk of STI acquisition and onward transmission than men who do not. They report high numbers of partners, but the minority are paid partners. They are an important core group in STI transmission.

## Introduction

Men who pay for sex (MPS) are considered to be a bridging population for sexually transmitted infections (STI), as their paid partners are often individuals at high STI risk, whose risk is conferred to the unpaid partners of MPS, with whom condom use is less likely.^1–4^ MPS may thus be at higher risk of STIs than men who do not pay for sex.^1^ ^5–7^ A study of Scottish genitourinary medicine (GUM) clinic attendees found that over half of paid sex occurred abroad, primarily in Europe and Asia,^8^ potentially driven by known ‘hotspot areas’ for sex tourism, such as Amsterdam or Bangkok.^9^ As MPS are a hard-to-reach population for research and intervention, most studies have used convenience sampling to access them,^10^ such that their data may not be representative of the general population. Britain's third National Survey of Sexual Attitudes and Lifestyles (Natsal-3) by contrast, uses probability sampling of the general population. Initial results from Natsal-3 show that 4% of men aged 16–44 years reported paying for sex in the 5 years prior to interview in 2010–2012, and that prevalence is similar to a decade ago.^11^ However, the role of this minority of the population in the transmission dynamics of STI/HIV depends on the prevalence of paid partners and on the number of paid and unpaid partners. Furthermore, as STI/HIV prevalence varies globally,^12^ it is also important to know in which global regions men pay for sex.

We examine variations in the prevalence of paying for sex and the recency of last paid sex by age among men in Britain, and estimate the proportion of paid sex partners among British men's sexual partners. We also explore how paying for sex varies by key sociodemographic, sexual behaviour, and health-seeking variables, including self-reported STI diagnosis/es, and how it is statistically associated with these variables. Last, we identify the global geographical regions where men pay for sex. Given that the prevalence of women paying for sex among 16–44 year olds in the past 5 years is 0.1%,^11^ the analyses presented in this paper focus exclusively on men.

## Methods

### Study design and participants

Natsal-3 is a stratified probability sample survey of 15 162 men and women (6293 men) aged 16–74 years, resident in Britain, undertaken between September 2010 and August 2012. Details of the sampling methodology and data collection are published elsewhere.^11^ ^13^ Briefly, participants were interviewed using a combination of face-to-face, computer-assisted, personal interviewing (CAPI) and computer-assisted self-interviewing (CASI). The more sensitive questions, including those on paying for sex and sex while outside the UK, were asked in the CASI.^13^ The response rate was 57.7% and the cooperation rate (ie, of all eligible addresses contacted) was 65.8%.

### Interviewing and data collection

Men were asked if they had ever paid for sex with a man and/or a woman. Those who reported they had paid for sex were then asked when they last paid for sex, how many sexual partners they had ever paid, and whether they had ever paid for sex while outside the UK. Men who had paid for sex while outside the UK were asked a further question about the geographical region in which they had paid for sex.^13^ Men were also asked to indicate whether or not they had included their paid partners in their total reported partners at the time of interview (referred to hereon as ‘lifetime partners’ for brevity). Demographic variables include age and relationship status at interview, individual socioeconomic status according to the National Statistics Socio-Economic Classification (NS-SEC),^14^ and area-level deprivation, using quintiles of the Index of Multiple Deprivation (IMD), a multidimensional measure combining income, employment, health, education, access to housing and services, crime and living environment.^15^ Measures of health, sexual health, and health-seeking behaviour include frequency of binge drinking (consuming more than eight units of alcohol on one occasion),^16^ recent drug use, low sexual function,^17^ ^18^ STI diagnoses, and sexual health clinic attendance. Finally, measures of sexual risk behaviour include number of sexual partners, reporting new foreign sexual partner(s) while outside the UK,^19^ same-sex experience, condom use, reporting concurrent partners, and finding sexual partners on the internet.

### Statistical analysis

Prevalence of paying for sex and recency of the occasions of the experience (ever, past 5 years, and past year) was examined by age group. Prevalence of same-sex paid sex, 0.2% (95% CI 0.1% to 0.4%) of the male population, was too low to conduct any separate analyses. The number of paid partners ever reported was added to the number of lifetime partners reported if the participant had indicated that these partners had not been included in their partner counts. Mean lifetime partner numbers, mean paid partner numbers, and the proportion of paid partners were calculated and examined across sociodemographic, general health, sexual behaviour, and sexual health-related variables for the population and for all MPS. The proportion of paid partners was calculated by dividing total reported paid partners by total lifetime partners for the population and within the categories of each variable. CIs were bootstrapped, as the resulting estimates were calculated from summary statistics and needed to have SEs estimated. Unadjusted logistic regression models were used to explore associations between reporting paying for sex in the 5 years prior to interview and sociodemographic, general health, and sexual behaviour variables. Multivariable logistic regressions adjusting for the potentially confounding effect of key sociodemographic variables were used to assess the associations of general health and sexual behaviour variables with the outcome of paying for sex in the past 5 years. Sexual behaviour variables were additionally adjusted for the confounding effect of the reported number of sexual partners in the past 5 years. Additional univariable and multivariable logistic regressions adjusting for sociodemographic variables and number of sexual partners in the past 5 years assessed the associations between paying for sex and key sexual health outcomes. Finally, we drew comparisons between the geographical regions where men had ever paid for sex when outside the UK, and the geographical origin of unpaid new sex partners while outside the UK, in the past 5 years. Those reporting unpaid sex partners outside the UK were restricted to those who had never paid for sex abroad to give a distribution of their partners’ origins without including any potential paid partners. No formal statistical comparison between the regional distributions for paying for sex abroad and having new sex partners abroad are made because of inconsistent time periods and subtle changes in wording in the questions of comparative interest.

All analysis used Stata V.12.1,^20^ and accounted for the sample weighting, clustering and stratification within the Natsal-3 sample. The data were first weighted to correct for unequal selection probabilities and then to match the demographic profile of the British population figures, in terms of gender, age and Government Office Region, according to the 2011 UK census.^13^

## Results

### Variation in prevalence by age

A total of 6108 men answered the question on paying for sex (97.1% of all male participants in Natsal-3), and of these, 11.0% (95% CI 10.1% to 11.9%) reported ever paying for sex; 3.6% (95% CI 3.1% to 4.2%) of all men reported paying for sex in the past 5 years, and 1.1% (95% CI 0.9% to 1.5%) reported doing so in the past year (table 1). Reporting ever paying for sex was lowest in men aged 16–24 years and three times as high in 25–34-year-olds. Lifetime prevalence was highest in men aged 55–64 years, while men aged 25–34 years had the highest prevalence in the past 5 years. Paid partners accounted for 4.6% (95% CI 4.5% to 4.7%) of all reported partners (table 1). This percentage ranged from 1.4% (95% CI 1.3% to 1.4%) of all partners reported by men aged 16–24 years to 7.5% (95% CI 7.3% to 7.8%) of all partners reported by men aged 55–64 years.

**Table 1:** Prevalence of paying for sex (ever, past 5 years, past year), mean partners numbers and percentage of total partnerships paid in the population by age group at interview

	Age group at interview (years)						
Timeframe	16–24	25–34	35–44	45–54	55–64	65–74	All
*Ever*							
Prevalence (%)	3.6	12.6	12.8	12.7	13.6	9.3	11.0
95% CI	(2.8 to 4.7)	(10.8 to 14.7)	(10.6 to 15.5)	(10.5 to 15.3)	(11.2 to 16.4)	(7.1 to 12.2)	(10.1 to 11.9)
Unweighted, weighted participants	1713, 1226	1495, 1346	793, 1401	767, 1367	728, 1134	612, 790	6108, 7265
Mean (SD) number of sexual partners	6.7 (13.4)	15.0 (25.6)	16.1 (33.1)	16.9 (28.6)	13.5 (29.8)	11.6 (36.0)	13.6 (28.4)
Mean (SD) number of paid sexual partners	0.1 (0.8)	0.5 (2.4)	0.6 (3.6)	0.9 (5.6)	1.0 (6.1)	0.7 (7.0)	0.6 (4.5)
Unweighted, weighted participants	1701, 1218	1471, 1327	787, 1387	750, 1345	700, 1091	586, 762	5995, 7130
Proportion of sexual partners paid (%)*	1.4	3.1	3.9	5.4	7.5	5.6	4.6
95% CI†	(1.3 to 1.4)	(3.0 to 3.1)	(3.8 to 4.1)	(5.2 to 5.7)	(7.3 to 7.8)	(5.3 to 6.0)	(4.5 to 4.7)
Unweighted, weighted partners	11429, 8232	22101, 21073	12705, 25442	12683, 24492	9454, 16318	6770, 9976	81391, 96445
*Past 5 years*							
Prevalence (%)	2.7	5.4	3.9	3.6	3.6	1.2	3.6
95% CI	(1.9 to 3.7)	(4.3 to 6.8)	(2.7 to 5.7)	(2.5 to 5.1)	(2.4 to 5.4)	(0.6 to 2.4)	(3.1 to 4.2)
Unweighted, weighted participants	1713, 1226	1495, 1346	793, 1401	767, 1367	728, 1134	612, 790	6108, 7265
*Past year*							
Prevalence (%)	0.8	1.2	1.2	1.1	1.8	0.4	1.1
95% CI	(0.4 to 1.4)	(0.8 to 2.0)	(0.6 to 2.3)	(0.5 to 2.2)	(1.0 to 3.3)	(0.1 to 1.2)	(0.9 to 1.5)
Unweighted, weighted participants	1713, 1226	1495, 1346	793, 1401	767, 1367	728, 1134	612, 790	6108, 7265

*Calculated as total paid partners divided by total lifetime partners for all men in each group.

†CI is estimated using 100 bootstrapped replications of each estimate.

### Proportion of all partnerships among MPS that are paid

On average, MPS reported more than twice as many lifetime partners as the male population average (means of 31.6 and 13.6, respectively) which holds true across all age groups (table 2). Among men who had ever paid for sex, one in five of their partners were paid, increasing with age from 12.8% of partners in men aged 16–24 years to 26.1% of partners in men aged 65–74 years. This proportion was higher among men who reported being in bad or very bad health, driven by a low denominator of mean lifetime partner numbers, and not the numerator of mean paid partner numbers. The proportion of paid partners was lower among men who had used drugs other than cannabis in the past year, driven by high mean lifetime partner numbers. While the prevalence of men paying for sex in the past 5 years was higher in men who reported partners outside the UK, same sex contact, finding partners on the internet, and concurrent partners, these groups did not report proportionally more paid partners than other men who paid for sex.

**Table 2:** Mean lifetime and paid partner numbers, and percentage of total partnerships paid among men who have ever paid for sex, by key demographic, general health, sexual behaviour and sexual health variables

	Mean (SD) number of lifetime sexual partners			Proportion of total partnerships paid*		
	Total	Paid	Participants†,‡	Per cent	95% CI§	Partners†
All men who ever paid for sex	31.6 (43.7)	5.8 (12.6)	616, 774	18.4	(18.2 to 18.7)	19465, 23956
*Demographics*						
Age group (years)						
16–24	20.1 (18.2)	2.6 (3.5)	60, 44	12.8	(12.4 to 13.3)	1203, 896
25–34	28.6 (32.6)	3.7 (5.9)	192, 166	13.4	(13.0 to 13.8)	5484, 4536
35–44	33.0 (45.4)	5.1 (8.8)	106, 176	14.9	(14.4 to 15.5)	3495, 6354
45–54	35.7 (43.7)	7.3 (13.9)	107, 173	20.6	(20.0 to 21.1)	3823, 6089
55–64	32.5 (48.5)	7.5 (15.1)	98, 147	23.5	(22.6 to 24.3)	3189, 4807
65–74	30.4 (58.8)	7.4 (22.3)	53, 68	26.1	(24.8 to 27.5)	1613, 2308
Relationship status at interview						
Living with a partner	29.8 (36.0)	5.1 (8.3)	321, 505	17.3	(17.0 to 17.6)	9579, 14938
In a steady, non-cohabiting relationship	31.8 (32.6)	4.1 (7.0)	69, 60	13.2	(12.5 to 13.9)	2196, 1720
No current steady partner	33.7 (53.5)	8.1 (20.1)	225, 208	23.6	(23.0 to 24.3)	7589, 6647
NS-SEC^14^ code (individual socioeconomic status)						
Semiroutine/routine	33.8 (52.4)	6.9 (17.9)	206, 233	20.2	(19.7 to 20.7)	6958, 7619
Intermediate	30.1 (40.3)	5.5 (9.2)	108, 135	19.3	(18.6 to 20.0)	3246, 4345
Managerial/professional	30.7 (34.5)	5.3 (9.8)	244, 343	17.3	(16.9 to 17.8)	7487, 10138
Full-time student	26.1 (34.5)	2.3 (2.4)	19, 19	10.5	(9.2 to 11.9)	495, 433
No job (10+ h/week) or not in past 10 years	24.0 (22.6)	6.4 (8.8)	36, 40	28.4	(27.2 to 29.7)	863, 849
Resident in Greater London						
No	31.5 (43.6)	5.5 (11.8)	533, 641	17.7	(17.4 to 18.0)	16790, 20150
Yes	32.1 (44.2)	7.1 (15.9)	83, 133	21.7	(20.8 to 22.7)	2662, 3703
Quintiles of Index of Multiple Deprivation**						
1 (least deprived)	31.9 (42.8)	5.3 (10.6)	130, 167	16.9	(16.3 to 17.5)	4141, 4939
2	24.3 (24.4)	3.5 (4.8)	110, 152	14.5	(13.9 to 15.0)	2673, 3431
3	31.9 (43.1)	5.9 (8.1)	123, 156	18.5	(17.8 to 19.1)	3928, 4892
4	35.4 (55.5)	7.3 (19.5)	112, 139	21.2	(20.3 to 22.1)	3963, 5033
5 (most deprived)	34.7 (46.8)	7.1 (15.2)	141, 159	20.5	(19.9 to 21.1)	4890, 5442
*General health*						
Self-reported health status						
Very good/good	31.0 (40.6)	5.7 (12.7)	496, 631	18.4	(18.0 to 18.7)	15397, 19130
Fair	38.1 (61.2)	6.7 (13.4)	92, 110	17.7	(17.0 to 18.4)	3508, 4121
Bad/very bad	18.2 (20.9)	5.5 (6.2)	27, 31	32.2	(30.4 to 33.9)	490, 610
Current frequency of binge drinking††						
Never/rarely	30.0 (45.3)	5.6 (10.3)	303, 416	18.9	(18.5 to 19.3)	9079, 11912
Monthly	30.8 (31.8)	4.3 (5.8)	112, 133	13.9	(13.5 to 14.4)	3449, 4047
Weekly/daily	37.2 (49.0)	7.3 (18.5)	171, 190	19.6	(19.0 to 20.2)	6357, 6972
Current smoking status						
Never smoked	27.9 (43.4)	6.5 (16.6)	220, 283	23.6	(23.1 to 24.2)	6135, 8129
Ex-smoker	31.4 (42.7)	5.6 (10.9)	178, 253	18.2	(17.7 to 18.8)	5597, 7495
Current smoker	36.2 (44.6)	5.1 (7.9)	218, 238	14.1	(13.7 to 14.5)	7885, 8160
Drug use, past year						
No	29.5 (45.6)	6.1 (13.8)	436, 591	20.9	(20.6 to 21.3)	12877, 17136
Yes, cannabis only	29.7 (27.1)	5.1 (9.9)	64, 66	18.0	(17.1 to 18.8)	1904, 1816
Yes, other hard drugs	43.1 (39.2)	4.4 (5.7)	116, 116	10.3	(9.9 to 10.7)	5005, 4663
*Sexual behaviour*						
New foreign sex partners abroad, past 5 years‡‡						
No	30.7 (45.7)	5.5 (11.7)	474, 610	18.2	(17.9 to 18.4)	14541, 18705
Yes	35.2 (35.0)	7.0 (15.7)	141, 163	19.4	(18.7 to 20.1)	4959, 5221
Same sex contact, past 5 years						
No	29.3 (40.1)	5.3 (11.8)	583, 726	18.3	(18.0 to 18.6)	17054, 20896
Yes	67.0 (71.6)	13.1 (20.0)	33, 48	19.9	(18.6 to 21.2)	2211, 3325
Used internet to find sexual partners, past year						
No	29.8 (41.9)	5.5 (12.5)	530, 680	18.7	(18.4 to 19.0)	15801, 20125
Yes	44.4 (52.8)	8.0 (13.5)	86, 95	17.7	(16.7 to 18.6)	3821, 3734
Used condom for HIV/STI prevention, past year						
No	30.5 (41.3)	5.4 (11.3)	453, 612	17.9	(17.6 to 18.2)	13802, 18657
Yes	29.6 (40.1)	6.0 (12.5)	151, 145	20.1	(19.5 to 20.7)	4471, 4331
Overlap between partners, past 5 years						
No	25.9 (37.8)	5.1 (10.8)	395, 524	20.0	(19.7 to 20.3)	10242, 12823
Yes	43.7 (52.1)	7.3 (15.7)	219, 248	16.6	(16.0 to 17.1)	9567, 10614
*Sexual health*						
Low sexual function, past year§§						
No	33.5 (45.4)	5.5 (10.8)	386, 489	16.3	(16.0 to 16.7)	12935, 15843
Yes	33.1 (43.2)	6.8 (13.1)	139, 182	20.9	(20.3 to 21.5)	4602, 6157
Sexual health clinic attendance, past 5 years						
No	28.9 (42.0)	5.5 (12.2)	484, 637	19.3	(19.0 to 19.6)	14008, 18677
Yes	44.1 (50.6)	7.2 (15.2)	117, 120	16.1	(15.5 to 16.8)	5157, 4468
HIV test, past 5 years						
No	29.8 (42.4)	5.4 (12.0)	457, 583	18.2	(17.9 to 18.6)	13622, 17207
Yes	40.2 (44.8)	6.8 (14.0)	125, 148	16.5	(15.9 to 17.1)	5029, 5378
STI diagnosis/es, past 5 years¶¶						
No	30.4 (41.8)	5.4 (11.8)	543, 690	17.9	(17.6 to 18.2)	16530, 20734
Yes	45.4 (60.2)	9.7 (19.1)	62, 69	21.2	(20.5 to 22.0)	2818, 2806

*Calculated as total paid partners divided by total lifetime partners for all men in each group.

†Unweighted, weighted denominators.

‡Small denominators (<30)^13^ may produce less reliable estimates for the population of MPS.

§CI is estimated using 100 bootstrapped replications of each estimate.

**Index of Multiple Deprivation (IMD) is a multidimensional measure of area (neighbourhood)-level deprivation based on the participant's postcode: IMD scores for England, Scotland and Wales were adjusted before being combined and assigned to quintiles, using a method by Payne and Abel.^15^

††More than 8 units at one time.^16^

‡‡Excludes partners from the UK while outside of the UK.

§§Sexual function score calculated using the Natsal-SF^17^ ^18^ only for the population who were sexually active the past year.

¶¶STIs include genital warts, trichomonas, gonorrhoea, chlamydia, syphilis, non-specific or non-gonococcal urethritis, and pelvic inflammatory disease.

MPS, men who pay for sex; NS-SEC, National Statistics Socio-Economic Classification; STI, sexually transmitted infection.

### Factors associated with paying for sex

After adjustment for age, relationship status, individual socioeconomic status, residence and area-level deprivation, paying for sex in the past 5 years was most commonly reported by men aged 25–34 years (table 3). Men aged 16–24 years and 64–74 years were least likely to report paying for sex in this time period. Compared with men who lived with a partner, men with no current steady partner were more likely to have paid for sex in the past 5 years (adjusted OR (AOR) 2.58 (95% CI 1.84 to 3.62)). Men in a managerial or professional occupation were more likely to have paid for sex than men in other occupations (AOR 1.88 (95% CI 1.27 to 2.79)). Men who reported binge drinking once a week or more, or using drugs other than cannabis in the past year, were more likely to report paying for sex in the past 5 years than those who did not report binge drinking or drug use (AOR 1.84 (95% CI 1.30 to 2.61) and AOR 5.01 (95% CI 3.38 to 7.42), respectively) (table 3). The odds of reporting paying for sex increased with the number of sexual partners reported in the past 5 years (AOR for 5+ partners; 31.50 (95% CI 18.69 to 53.09) relative to reporting 0–2 partners). After additional adjustment for partner numbers, men who reported new foreign sexual partners while outside the UK, sexual partners found online, and concurrent partners, were also more likely to have paid for sex (AORs of 7.96 (95% CI 4.97 to 12.73), 1.97 (95% CI 1.28 to 3.03), and 2.47 (95% CI 1.60 to 3.80), respectively), while there was no association with using condoms specifically for HIV/STI prevention (AOR 1.40 (95% CI 0.93 to 2.10)).

**Table 3:** Variations in the prevalence of paying for sex in the past 5 years by key demographic, general health, and sexual behaviour factors

	Paid for sex, past 5 years		Univariable logistic regression			Multivariable logistic regression			
	Per cent	95% CI	OR	95% CI	p Value	AOR*	95% CI	p Value	Denominators†
All men	3.6	(3.1 to 4.2)	–	–	–	–	–	–	6108, 7265
*Demographics*									
Age group (years)					0.0002			<0.0001	
16–24	2.7	(1.9 to 3.7)	1.00			1.00			1713, 1226
25–34	5.4	(4.3 to 6.8)	2.05	(1.37 to 3.07)		3.13	(2.05 to 4.78)		1495, 1346
35–44	3.9	(2.7 to 5.7)	1.47	(0.88 to 2.46)		2.75	(1.60 to 4.72)		793, 1401
45–54	3.6	(2.5 to 5.1)	1.34	(0.82 to 2.20)		2.45	(1.47 to 4.10)		767, 1367
55–64	3.6	(2.4 to 5.4)	1.37	(0.81 to 2.30)		2.32	(1.34 to 4.00)		728, 1134
65–74	1.2	(0.6 to 2.4)	0.45	(0.22 to 0.95)		0.83	(0.39 to 1.77)		612, 790
Relationship status at interview					<0.0001			<0.0001	
Living with a partner	2.5	(2.0 to 3.2)	1.00			1.00			2969, 4710
In a steady, non-cohabiting relationship	3.6	(2.4 to 5.2)	1.43	(0.90 to 2.29)		1.45	(0.89 to 2.35)		959, 768
No current steady partner	6.3	(5.2 to 7.6)	2.59	(1.88 to 3.56)		2.58	(1.84 to 3.62)		2146, 1754
NS-SEC^14^ code (individual socioeconomic status)					0.1014			0.0001	
Semiroutine/routine	3.1	(2.3 to 4.0)	1.00			1.00			2018, 2319
Intermediate	3.5	(2.4 to 4.9)	1.13	(0.71 to 1.79)		1.34	(0.84 to 2.16)		913, 1202
Managerial/professional	4.5	(3.5 to 5.7)	1.49	(1.02 to 2.17)		1.88	(1.27 to 2.79)		1842, 2580
Full-time student	1.9	(1.0 to 3.8)	0.62	(0.29 to 1.30)		0.33	(0.15 to 0.73)		869, 660
No job (10+ h/week) or not in past 10 years	3.7	(2.2 to 6.3)	1.22	(0.66 to 2.26)		1.03	(0.54 to 1.97)		445, 477
Resident in Greater London					0.0963			0.1223	
No	3.4	(2.9 to 4.0)	1.00			1.00			5475, 6298
Yes	4.9	(3.3 to 7.3)	1.47	(0.93 to 2.30)		1.44	(0.91 to 2.29)		633, 967
Quintiles of Index of Multiple Deprivation§					0.7537			0.9233	
1 (least deprived)	3.2	(2.3 to 4.4)	1.00			1.00			1192, 1493
2	3.5	(2.4 to 5.1)	1.12	(0.67 to 1.87)		1.09	(0.65 to 1.83)		1207, 1533
3	3.7	(2.7 to 5.0)	1.16	(0.71 to 1.88)		1.02	(0.62 to 1.66)		1187, 1416
4	3.4	(2.4 to 4.6)	1.06	(0.66 to 1.71)		0.89	(0.54 to 1.46)		1225, 1453
5 (most deprived)	4.3	(3.1 to 5.8)	1.35	(0.85 to 2.15)		1.09	(0.69 to 1.72)		1297, 1370
*General health*									
Self-reported health status					0.1374			0.0323	
Very good/good	3.8	(3.2 to 4.4)	1.00			1.00			5027, 5935
Fair	3.2	(2.2 to 4.5)	0.84	(0.56 to 1.27)		0.72	(0.46 to 1.12)		844, 1042
Bad/very bad	1.6	(0.7 to 3.8)	0.42	(0.17 to 1.03)		0.33	(0.13 to 0.83)		235, 285
Current frequency of binge drinking¶					0.0015			0.0029	
Never/rarely	3.0	(2.4 to 3.8)	1.00			1.00			3398, 4242
Monthly	3.9	(2.8 to 5.6)	1.31	(0.86 to 1.99)		1.39	(0.90 to 2.14)		1026, 1132
Weekly/daily	5.5	(4.3 to 7.1)	1.88	(1.34 to 2.65)		1.84	(1.30 to 2.61)		1252, 1412
Current smoking status					0.0573			0.2054	
Never smoked	3.2	(2.5 to 4.0)	1.00			1.00			2958, 3434
Ex-smoker	3.2	(2.3 to 4.4)	1.01	(0.67 to 1.52)		1.16	(0.74 to 1.84)		1376, 1918
Current smoker	4.6	(3.6 to 5.9)	1.46	(1.04 to 2.07)		1.39	(0.96 to 2.01)		1774, 1914
Drug use, past year					<0.0001			<0.0001	
No	2.9	(2.4 to 3.4)	1.00			1.00			4765, 6035
Yes, cannabis only	3.4	(2.1 to 5.4)	1.21	(0.72 to 2.02)		1.08	(0.65 to 1.80)		663, 621
Yes, other hard drugs	14.1	(10.8 to 18.2)	5.60	(3.93 to 7.98)		5.01	(3.38 to 7.42)		517, 475
*Sexual behaviour*									
Number of sexual partners, past 5 years					<0.0001			<0.0001	
0–2	1.0	(0.7 to 1.3)	1.00			1.00			4136, 5442
3–4	6.3	(4.5 to 8.7)	6.79	(4.19 to 11.01)		10.27	(5.90 to 17.87)		758, 727
5+	15.7	(13.1 to 18.7)	18.94	(12.79 to 28.06)		31.50	(18.69 to 53.09)		1158, 1017
New foreign sex partners abroad, past 5 years**,††					<0.0001			<0.0001	
No	1.9	(1.6 to 2.4)	1.00			1.00			5662, 6781
Yes	29.9	(24.6 to 35.7)	21.60	(15.31 to 30.48)		7.96	(4.97 to 12.73)		417, 433
Same sex contact, past 5 years††					<0.0001			0.3145	
No	3.3	(2.8 to 3.8)	1.00			1.00			5904, 7058
Yes	14.2	(8.8 to 22.3)	4.91	(2.79 to 8.62)		1.36	(0.75 to 2.45)		203, 206
Used Internet to find sexual partners, past year††					<0.0001			0.0021	
No	3.0	(2.5 to 3.5)	1.00			1.00			5290, 6574
Yes	18.1	(13.6 to 23.6)	7.21	(4.96 to 10.49)		1.97	(1.28 to 3.03)		389, 362
Used condom for HIV/STI prevention, past year††					<0.0001			0.1066	
No	2.7	(2.2 to 3.3)	1.00			1.00			4230, 5673
Yes	8.9	(7.2 to 11.0)	3.49	(2.58 to 4.73)		1.40	(0.93 to 2.10)		1279, 1081
Overlap between partners, past 5 years††					<0.0001			<0.0001	
No	1.7	(1.3 to 2.1)	1.00			1.00			5005, 6129
Yes	14.6	(12.1 to 17.5)	9.99	(7.29 to 13.68)		2.47	(1.60 to 3.80)		1057, 1065

*ORs adjusted for age (continuous), ‘relationship status at interview’, ‘NS-SEC code’, ‘resident in Greater London’, and ‘quintiles of multiple deprivation’. Variables are not adjusted for themselves. ‘Age group’ is not adjusted for age (continuous).

†Unweighted, weighted denominators.

§Index of Multiple Deprivation (IMD) is a multidimensional measure of area (neighbourhood)-level deprivation based on the participant's postcode: IMD scores for England, Scotland and Wales were adjusted before being combined and assigned to quintiles, using a method by Payne and Abel.^15^

¶More than 8 units at one time.^16^

**Excludes partners from the UK while outside the UK.

††Additionally adjusted for ‘number of sexual partners, past 5 years’.

AOR, adjusted OR; NS-SEC, National Statistics Socio-Economic Classification; STI, sexually transmitted infection.

### Sexual health outcomes associated with paying for sex

In the past 5 years, partners of MPS accounted for 14.7% of all reported sexual partners, and MPS accounted for 15.6% of all men reporting STI diagnoses (data not shown). After adjusting for the number of sexual partners, MPS were still more likely to report the sexual health outcomes listed in table 4 than those who did not pay for sex; they were more likely to report STI diagnosis/es in the past 5 years (AOR 2.34 (95% CI 1.44 to 3.81)) and to have lower sexual function in the past year (AOR 1.80 (95% CI 1.25 to 2.69)).^17^ These men were also more likely to report having attended a sexual health clinic (AOR 1.69 (95% CI 1.10 to 2.58)) and testing for HIV (AOR 2.20 (95% CI 1.53 to 3.17)) in the past 5 years.

**Table 4:** Variations in the prevalence of sexual health outcomes by paying for sex in the past 5 years

	No paid sex		Paid for sex, past 5 years		Univariable logistic regression			Multivariable logistic regression		
Unweighted, weighted denominators	5872, 7004		236, 261							
	Per cent	95% CI	Per cent	95% CI	OR	95% CI	p Value	AOR^*^	95% CI	p Value
Low sexual function, past year†							0.0016			0.0037
No	80.4	(79.1 to 81.8)	69.6	(61.6 to 76.5)	1.00			1.00		
Yes	19.6	(18.2 to 20.9)	30.4	(23.5 to 38.4)	1.80	(1.25 to 2.59)		1.81	(1.21 to 2.69)	
Sexual health clinic attendance‡							<0.0001			0.0166
No	89.2	(88.3 to 90.0)	69.8	(62.2 to 76.4)	1.00			1.00		
Yes	10.8	(10.0 to 11.7)	30.2	(23.6 to 37.8)	3.58	(2.52 to 5.07)		1.69	(1.10 to 2.58)	
HIV test‡							<0.0001			<0.0001
No	89.5	(88.5 to 90.4)	65.6	(57.6 to 72.8)	1.00			1.00		
Yes	10.5	(9.6 to 11.5)	34.4	(27.2 to 42.4)	4.49	(3.15 to 6.39)		2.20	(1.53 to 3.17)	
STI diagnosis/es‡,§							<0.0001			0.0007
No	96.9	(96.4 to 97.3)	83.6	(77.4 to 88.3)	1.00			1.00		
Yes	3.1	(2.7 to 3.6)	16.4	(11.7 to 22.6)	6.07	(3.93 to 9.38)		2.34	(1.44 to 3.81)	

*ORs for paying for sex are adjusted for age (continuous), ‘relationship status at interview’, ‘NS-SEC code’, ‘resident in Greater London’, ‘quintiles of multiple deprivation’, and ‘number of sexual partners, past 5 years’.

†Sexual function score calculated using the Natsal-SF^17^ ^18^ only for the population who were sexually active in the past year.

‡In the past 5 years.

§STIs include genital warts, trichomonas, gonorrhoea, chlamydia, syphilis, non-specific or non-gonococcal urethritis and pelvic inflammatory disease.

AOR, adjusted OR; NS-SEC, National Statistics Socio-Economic Classification; STI, sexually transmitted infection.

### Where men pay for sex

Among men who reported ever paying for sex, 62.6% (95% CI 58.3% to 66.8%) had, at some time, paid for sex outside the UK (data not shown). The most commonly reported geographic regions where men paid for sex were Europe (64.5% (95% CI 58.9% to 69.6%)) and Asia (25.4% (95% CI 20.9% to 30.5%)), with other regions accounting for much smaller proportions (see web appendix). Among men who had *not* paid for sex, Europe (52.7% (95% CI 45.9% to 59.4%)) and Asia (13.9% (95% CI 9.8% to 19.2%)) were also the most commonly reported geographic regions in terms of the *origins* of their new foreign (unpaid) partners. By contrast with men who had paid for sex, large proportions of men who had *not* paid for sex also reported having sex abroad with people who usually lived in North America (22.1% (95% CI 16.9% to 28.5%)) and Australasia (12.4% (95% CI 8.5% to 17.7%)).

## Discussion

Around one in 10 men in Britain report having ever paid for sex. These men account for a disproportionate number of sexual partners reported by all men, of which the minority (18.4%) are paid. They are also more likely to report STI diagnoses even when accounting for their disproportionately larger number of sexual partners, which is frequently considered the most important behavioural variable associated with STI diagnoses.^21^ This evidence strongly supports the idea that these men are a bridge for disassortative sexual mixing and for the spread of STIs. MPS are most likely to be aged between 25 and 34 years, single, in managerial or professional occupations, and have high partner numbers. After adjusting for the key risk behaviour of sexual partner numbers, these men still report many other sexual behaviours, such as having new foreign partners while outside the UK, and with less pronounced differences in sexual health-related behaviour, such as STI clinic attendance or condom use, exposing an increased vulnerability without an increase in precautionary behaviour. However, this increase in precautionary behaviour, whether it be a consequence of other sexual behaviour or not, may present an opportunity for targeted interventions for these men.

Despite lifetime prevalence of paid sex varying little between men aged 25–64 years, there is a steady increase with age in the mean number of paid partners, suggesting either generational changes in paid sex, or that a proportion of MPS continue to pay for sex as they age. The varying proportions of partners paid among MPS is largely driven by *lifetime* partner numbers rather than numbers of *paid* partners, such that a lower proportion of paid partners actually reflects a high number of lifetime partners. However, for some reported behaviours, such as sex partners outside the UK, same sex contact, sex partners found online, and concurrent partners, total and paid partner numbers increase. This suggests that MPS exhibiting these behaviours have higher lifetime partner numbers than other MPS as well as higher paid partner numbers, putting them at a higher risk for STIs than other MPS.

The theory of Situational Sexual Behaviour may provide some explanation for the strong relationships observed between reporting paying for sex and new sex partners while outside the UK. The theory posits that sexual behaviour which is different to an individual's normal sexual behaviour, like paying for sex, is sometimes determined by the set of circumstances in which individuals find themselves.^22–24^ For example, as a place removed from the day-to-day activities of most men, overseas travel facilitates the opportunity for paid sex. These opportunities may also be facilitated by an ease of access in terms of proximity or cost of travel to an area with an established sex-work industry, and/or a legislative framework which serves to increase the safety of the encounter, as in The Netherlands.^25^

As paying for sex in the past 5 years was far more commonly reported by men who had new sexual partners while outside the UK (29.9%), it is then not surprising that the majority of men who had paid for sex reported doing so outside the UK, with Europe and Asia the most commonly reported geographical regions. It is interesting to consider these geographical patterns in the context of Britons’ international travel patterns with nearly 80% of British residents visiting Europe when travelling abroad in 2011, and around half of all paid sex outside the UK occurring in Europe. By contrast, a fifth of all paid sex outside the UK occurred in Asia, but this is the destination for only 1 in 20 Britons travelling abroad.^26^ This suggests that travel to Asia results in disproportionately more sex tourism than travel to Europe. Although paid sex in South America and Australasia constitutes around 5% of reported paid sex, only 1% of travel is to these destinations, suggesting a similar imbalance between sex tourism and travel. These findings concur with those of earlier studies,^11^ ^12^ and have implications for targeting sexual health interventions and health promotion messages to those travelling to these sex tourism areas, especially given the higher STI/HIV prevalence in these regions.^10^

As with all cross-sectional studies, causality cannot be determined. This is especially problematic with data relating to sexual risk and health-seeking behaviour, as we can only assume that increased sexual risk resulted in increased health-seeking behaviour. Questions referring to an entire sexual history, such as lifetime partners, rather than more recent timeframes, may be subject to recall biases. There are also likely to be different types of MPS, and it is possible that our data may be skewed by particular types of MPS. As a population-based survey of a wide range of sexual behaviours, Natsal-3 asked a limited number of questions about paying for sex. As a result, it was not possible to determine whether men paid for sex in the UK only, outside the UK only, or in and outside the UK. This also means that we are not able to measure the extent of overlap between reporting paying for sex and reporting new sex partners outside the UK. Additionally, formal statistical comparisons across the geographical regions for paid and unpaid partners are not possible because of differences in timeframes and wording of the questions of comparative interest, that is, *origin* of unpaid partners abroad and *region where* paid partners were found. Last, the geographical regions listed in the questionnaire where paid sex occurred are very large, and future surveys should consider asking men for specific locations which could subsequently be categorised.

Despite these limitations, data from Natsal-3 enable us to present the first ever population-based estimates of where British men pay for sex outside the UK, and update our understanding of the factors associated with paying for sex using data that are broadly representative of the British population. Our data show that MPS continue to be at increased risk of STI acquisition and onward transmission. Further research is needed to establish why men may choose to pay for sex in their own country, while abroad, or both, and whether these groups of men and their behaviours are heterogeneous and, if so, how.

**Key messages**

- Men who pay for sex are at higher risk of acquiring STIs even after considering other key STI risk behaviours.
- Having paid partners may not directly increase men's STI risk, but may rather be a marker for men who are likely to partake in other sexual risk behaviours.
- Men who pay for sex should be considered a core-group for sexual health interventions and services.

## Supplementary Material

Web supplement
