# Supplementary material for: The prevalence of, and factors associated with, paying for sex among men resident in Britain: findings from the third National Survey of Sexual Attitudes and Lifestyles (Natsal-3)
Source: Sex Transm Infect. 2014 Nov 17;91(2):116–23. doi: 10.1136/sextrans-2014-051683 (PMC4345903; doi:10.1136/sextrans-2014-051683)
Supplement: Web supplement [file sextrans-2014-051683-s1.pdf]

Web appendix: Geographic region where ever paid for sex and geographic origin of unpaid partners while outside the UK in the past 5 years

	Of all men who had sex outside the UK			
	Region where paid for sex while outside the UK, ever		Origin of unpaid sex partners while outside the UK, past 5 years[1]	
Unweighted, weighted denominators	*387, 498*		*292, 289*	
	**Percent**	**95% C.I**	**Percent**	**95% C.I**
**EU (not incl. UK)**	64.5%	(58.9-69.6)	52.7%	(45.9-59.4)
**Asia**	25.4%	(20.9-30.5)	13.9%	(9.8-19.2)
**Sub-Saharan Africa**	7.0%	(4.5-10.8)	5.8%	(3.2-10.2)
**South America**	6.6%	(4.4-9.8)	5.8%	(3.4-9.5)
**Middle East/North Africa**	5.2%	(3.2-8.5)	2.8%	(1.1-6.7)
**Australasia**	5.3%	(3.0-9.0)	12.4%	(8.5-17.7)
**North America**	5.0%	(2.9-8.3)	22.1%	(16.9-28.5)
**Caribbean**	3.2%	(1.7-5.8)	1.1%	(0.3-3.9)

[1] Excludes 121 (140 weighted) men who report paying for sex abroad to avoid counting any paid partners and biasing the data towards the regional distribution of paid partners.
[2] Columns do not add to 100% as men can report more than one region
